# Temperature and Health

**Published:** 1899-08-05

**Authors:** 


### The Hospital

A Journal of

The Medical Sciences and Hospital Administration.

Vol. XXVI.—No. 671.] Edited by Sir Henry Burdett, K.C.B., and Solomon C. Smith, M.D., M.R.C.P. [August 5, 1899.

### Temperature and Health.

The prevalence of high temperature over the greater portion of the United Kingdom may fairly be described as an event which, during some period at least of the months of July or August, is of annual occurrence, and which, nevertheless, seems always to be regarded as unforeseen and abnormal. Year after year it is written and talked about as if the weather had never before been sultry, and year after year the columns of our newspapers are supplied by correspondents with amazing records of impossible temperatures, furnished by idiosyncratic or imperfectly sheltered thermometers. Year after year there is the same indifference to the experience which teaches that a brief and sudden prevalence of heat calls for a corresponding modification of ordinary conditions of living and working, and year after year there is the same list of casualties which attention to this experience would have obviated. We read of "sunstroke" or "heat-apoplexy" as occurring not only in persons who are chained to the routine of some humble calling, but even to those who are enabled by the possession of means and leisure to adapt themselves without difficulty to varying climatic conditions. Respectable citizens rush about the streets in heavy black hats and in thick, dark clothing, with red and perspiring faces, or they devote themselves to the morning newspaper until they are compelled to hurry for the accustomed train. They are always unprepared for what is certain to occur. When the thermometer approaches 80 degrees they have no cool clothing, and they think it useless to order any, because the great heat would be over before the tailor could send it home. The heavy meals, which are probably excessive even in the winter, are still consumed as a matter of habit, and the thirst incidental to excessive perspiration is quenched by large quantities of beverages which often contain a considerable amount of alcohol under the most insidious disguises. Office and business hours remain unchanged; and an employer who wished his workpeople to begin at four in the morning, and to leave off in the hotter parts of the day for a corresponding period of siesta, would call down upon his devoted head all the terrors of the worst forms of trades-unionism. Ruin would be too mild a punishment for a manufacturer who dared to endeavour to protect the health of "labour," without having first obtained the consent of some of its "leaders," and their consent, even if not absolutely withheld from a proposal emanating from "capital," would certainly not be obtained until the occasion for acting upon it had passed away.

For us, who profess to be a practical people, all this seems to be inexpressibly absurd; and our prevailing customs in hot weather would be regarded as insufferable, if they were imposed upon us by authority instead of by usage. And yet the majority continue in the old grooves, accepting our July and August as if they were visitations the severity of which we were unable to mitigate, and gasping and complaining as if these were our sole resources against an unexpected and overwhelming calamity. Our cousins and brothers, and even many of our sisters, are living in hot climates, are ruling or teaching tropical races, are bearing "the white man's burden" under all conceivable conditions, and are showing in their daily lives that heat can be rendered harmless by the concession of its reasonable demands. Science is beginning to teach that many of the diseases formerly ascribed to the influence of high temperature are really due to the operation of other causes, even if these be such that the temperature is either necessary or favourable to their presence and their activity. The mere heat is harmless, if we will consent to act in conformity with the physiological requirements of its effects upon the animal body. Why should not custom ordain that, in England, when the thermometer reaches some given elevation, say 75 degrees, social and business customs should be modified to meet the change? "Fashion," this year, early ordained that "there were to be no white hats," and the foreign visitor must have been either amused or puzzled at the head-gear of our well-dressed males during the last week or two. Why should not even Fashion submit to a temporary dethronement before the altar of Fahrenheit? Why should not paterfamilias say to himself in April, or in May, "Summer will soon be here, and I will provide myself with clothing suitable for it, so as to be prepared when it comes"? Why should not the wearers of broadcloth universally adopt loosely-fitting and light-coloured suits, and white hats, for the days of July and August, purely as a matter of course, and just as they would wear evening dress for dinner?

And why should not the dwelling-rooms be kept dark and cool, with closed windows and lowered blinds, until the evening and night, when the windows might be wide open and air be admitted freely? And why, under a sliding scale controlled by the inexorable thermometer, should not the hours of business admit of temporary modification? Or, perhaps most important of all, why should not the diet and the drinks be adapted to August rather than to November? Most of the illness, and nearly all the discomfort, which are incidental to heat might be obviated by reasonable precautions in this direction alone. A moderate but sufficient amount of meat, fish, and fat, with plenty of fruit and green vegetables, and an almost complete abandonment of the starch elements of common food, together with a decrease in the accustomed quantity of alcohol and an increase in the degree of its dilution, would render many of those who now suffer under high temperature almost unconscious of it as an inconvenience.

